# The Role of Nanoengineered Biochar Activated with Fe for Sulfanilamide Removal from Soils and Water

**DOI:** 10.3390/molecules27217418

**Published:** 2022-11-01

**Authors:** Beatriz Gámiz, Pilar Velarde, Kurt A. Spokas, Lucía Cox

**Affiliations:** 1 Instituto de Recursos Naturales y Agrobiología de Sevilla (IRNAS), CSIC, Avenida Reina Mercedes 10, 41012 Seville, Spain; 2 U.S. Department of Agriculture, Agricultural Research Service, 439 Borlaug Hall, 1991 Upper Buford Circle, St. Paul, MN 55108, USA

**Keywords:** activated biochar, antibiotics, biodegradation, bound residues, leaching, nanostructured materials, soil amendments

## Abstract

Biochar is a nanoengineered sorbent proposed to control the contamination derived from the presence of residual concentrations of sulfonamides in soil. In this work, we evaluated the sorption of sulfanilamide (SFA) in commercial biochar (BC) produced at 500 °C from oak hardwood (*Quercus ilex*) and its analog activated with 2% (*w*/*w*) Fe (BC-Fe). Subsequently, the effect on dissipation and transport of SFA in untreated soil and soil treated with BC and BC-Fe was also assessed. Laboratory batch studies revealed that BC-Fe increased the sorption of SFA as compared to the pristine BC with K_d_ of 278 and 98 L/kg, respectively. The dissipation of SFA in either untreated soil or soil treated with BC or BC-Fe was similar, displaying half-lives ranging between 4 and 6.4 days. Conversely, the concurrent determination of sorption during the incubation experiment showed that lower amounts of SFA in solution at the beginning of the experiments were bioavailable in BC-Fe-treated soil when compared to the rest of the treatments shortly after application. Leaching column studies confirmed the amendment’s capability to bind the SFA compound. Therefore, the decrease in bioavailability and movement of SFA in treated soils suggest that biochar soil application can reduce SFA soil and water contamination. According to our results, BC surface modification after Fe activation may be more appropriate for water decontamination than for soil since there were no significant differences between the two types of biochar when added to the soil. Therefore, these outcomes should be considered to optimize the SFA mitigation potential of biochar.

## 1. Introduction

The intensification of agricultural and livestock activity has been gradually increasing the use of veterinary antibiotics, thus bolstering public health risks and environmental problems that directly affect our society [1,2]. Sulfonamides are among the most common antibiotics used worldwide [3]. Antibiotics are released into the environment via their use in cattle rearing by soil fertilization with animal urine or manure and human use through sewage sludge or wastewater utilization [4,5,6]. Sulfonamide soil residues have been measured to be as high as several milligrams per kilogram in agricultural soils [7], which can produce problems for human health by entering the food chain [7,8,9]. An additional problem with these compounds is the progressive existence of antibiotic-resistant bacteria that are then more difficult to combat therapeutically [8,10].

This situation has stimulated the development of economical and effective mitigation methods for antibiotic removal from the environment [11,12]. Therefore, the sorption and persistence of sulfonamides need to be assessed since these are key factors determining their adverse impact [13,14]. The mobility of sulfonamides in soils has been studied by several authors in the last decades since sulfonamides generally possess high water solubilities and low affinity for solid surfaces [6,15]. Spielmeyer et al. [16] assessed the presence and movement of several sulfonamide antibiotics in a long-term study and observed a continuous presence of sulfonamides in leachates. They concluded that the presence of sorbed sulfonamides results in long-term leaching into deeper soil layers and groundwater contamination.

To control the contamination by antibiotics, several approaches have been proposed, which are grouped into destructive and non-destructive [17]. Among destructive methods, biodegradation, supported by abiotic reactions (e.g., chemical oxidation, precipitation, photocatalysis) [12], has been recently encouraged owing to their inexpensive nature and effective mitigation of sulfonamide-contaminated soil and water [3]. On the other hand, non-destructive methods, mainly consisting of sorption, are the most widespread, and the use of carbonaceous materials such as biochar as the sorbent is being highly promoted [18].

Biochar (BC) is the solid form of carbon resulting from the thermal treatment of biomass in a limited oxygen atmosphere. BC has gained growing interest due to its diverse physicochemical characteristics and miscellaneous applications in several areas, such as climate change mitigation, agriculture, environmental remediation, and energy production [19]. Hence, pyrolysis of biomass can potentially be a method of choice for both sustainable management of agricultural soils and as a cost-efficient sorbent for the removal of many contaminants from water and soil [20], such as heavy metals [21], dyes [22], or pesticides [23]. Recently, BC has also been receiving major attention as an effective sorbent for antibiotics [24,25,26]. The capability of BC to sorb organic contaminants is governed by its physicochemical properties, which include composition, porosity, surface area, functional groups, and pH [27]. Activation processes are progressively being adopted to further enhance the sorption capacity of BC as a methodology for soil remediation for organic pollutants and heavy metals [28,29,30]. The activation is advantageous for increasing the sorption of agrochemicals by low-temperature biochar (<500 °C). The interest in low-temperature biochar, for its lower sorption capacities than high-temperature biochar, lies in their suitability for soil fertility applications, since low-temperature BCs tend to have more labile organic substances and more O-functional groups than high-temperature biochar, which can act as nutrient exchange sites after oxidation [31,32,33].

Traditionally, pesticide sorption on biochar generally leads to a lower risk of leaching and added protection against biotic and abiotic degradation processes [34,35]. Hence, it is expected that the use of BC could reduce the bioavailability of antibiotics in soils, which would be beneficial to control microbial resistance and contamination problems. The activation of BC has been proposed as a sustainable solution for the removal of antibiotics from water [12]; however, the effect of BC activation on the fate of sulfonamides in soil is still in an early stage. The use of Fe as an activation agent has been extensively proposed to increase the sorption performance of regular biochar in water [36,37]. Recently, Bai et al. [38] found that magnetite-coated biochar enhanced the sorption and also promoted the redox transformation of the antibiotic sulfamethazine in water. However, little is known regarding the sorption performance of activated BC when it is added to the soil and even less when activated with Fe. Additionally, tackling specific soil constraints is cited as a current research need. There is limited information regarding BC’s effect on SFA’s soil fate, with most of the studies with BC and these compounds dealing with their direct water removal [38,39]. Another innovative feature assessed here was to ascertain whether the addition of a commercial BC and its activation with Fe induce modifications in the easily available fraction of sulfanilamide (SFA) during its dissipation in the soil and, eventually, to appraise its impact on the mobility of SFA through the soil profile as a strategy to protect water resources and soil quality. Hence, the goal of this study was to assess the sorption, dissipation, and transport of SFA in soil after its treatment with a commercial BC and its Fe-treated analog. Results from this work will be a major advancement in understanding the role of biochar as a strategy to control soil and water pollution.

## 2. Results and Discussion

### 2.1. Characterization of Biochar

Table 1 compiles the main characteristics of BC and BC-Fe. The most important feature to highlight was that both C and O content increased in the case of BC-Fe, which was the consequence of the oxidation of the material. Additionally, the Fe content was enhanced after the activation treatment from 2.7 g/kg for BC up to 43.9 g/kg for BC-Fe (Table 1).

**Figure 1:**
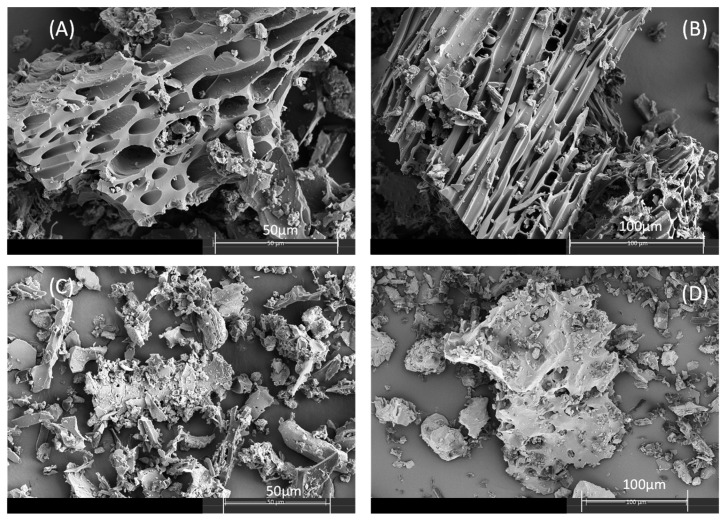
SEM visualization of individual biochar sample, (**A**,**B**) photographs correspond to BC and (**C**,**D**) with BC-Fe.

Regarding the specific surface areas, the SSA values were very low, although a slight increase upon the activation with Fe was observed. This could be attributed to the low size and/or poor crystallinity of the Fe species incorporated on the surface of the biochar [40]. Similarly, an increase in the micropore volume for BC-Fe was also observed, which agrees with the finer texture observed in the SEM images (Figure 1). The most remarkable feature of the microphotographs was the loss of big channels (mesopores) from the pristine BC (Figure 1, Table 1), which could be a result of the surface coating from the Fe-solution, reducing the total pore volume [40,41,42].

**Figure 2:**
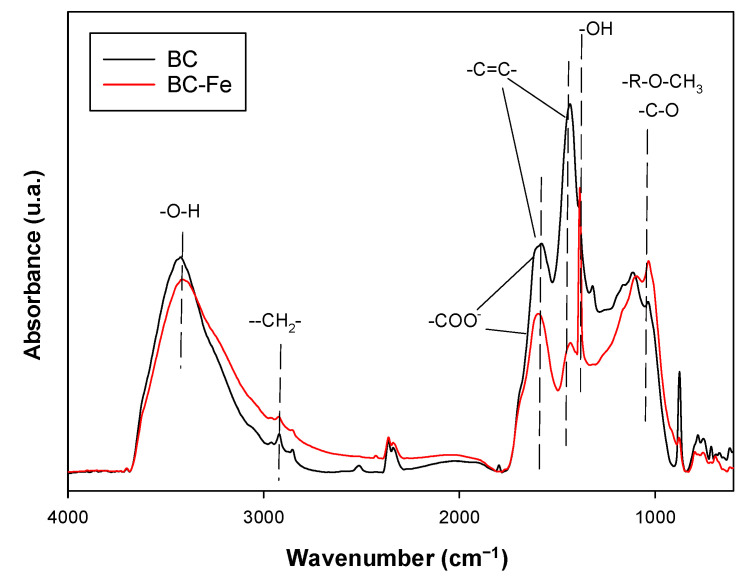
FTIR spectra for BC and BC-Fe indicating the most relevant bands recorded.

The FT-IR spectra of BC and BC-Fe are shown in Figure 2. In the unactivated BC, the band that appears at 3422 cm^−1^ is assigned to the vibration of -O-H bonds. Those recorded at 2918 and 2846 cm^−1^ correspond to aliphatic groups -C-H. The 1430 and 1600 cm^−1^ bands are attributed to strain vibrations -C=C- of aromatic rings, although the 1600 cm^−1^ band can also overlap the tension modes of carbonyl groups -C=O of conjugated ketones and quinones, as well as with the deprotonated form of the carboxylic group (-COO^−^). The peak at 872 cm^−1^ was endorsed as aromatic C-H as well as C-O from carbonates [43]. In the activated biochar (BC-Fe), most of these bands were also observed, indicating that it maintains most of the functional groups of the untreated BC. However, some characteristic bands, such as those at 1600 cm^−1^, 1430 cm^−1^, and 872 cm^−1^, became weaker, whereas a noticeable increase was observed in the 1384 cm^−1^ band assigned to O-H- bending of phenols and the band at 1032 cm^−1^, in which characteristics of acid derivatives, aliphatic C-O-C, and -OH from oxygenated functional groups of cellulose and hemicellulose and methoxy groups of lignin [44] were recorded. These changes are indicative of surface alterations due to the Fe- treatment and are likely related to the oxidation of the surfaces [44,45].

### 2.2. Sorption-Desorption Isotherms of SFA on Biochar

**Figure 3:**
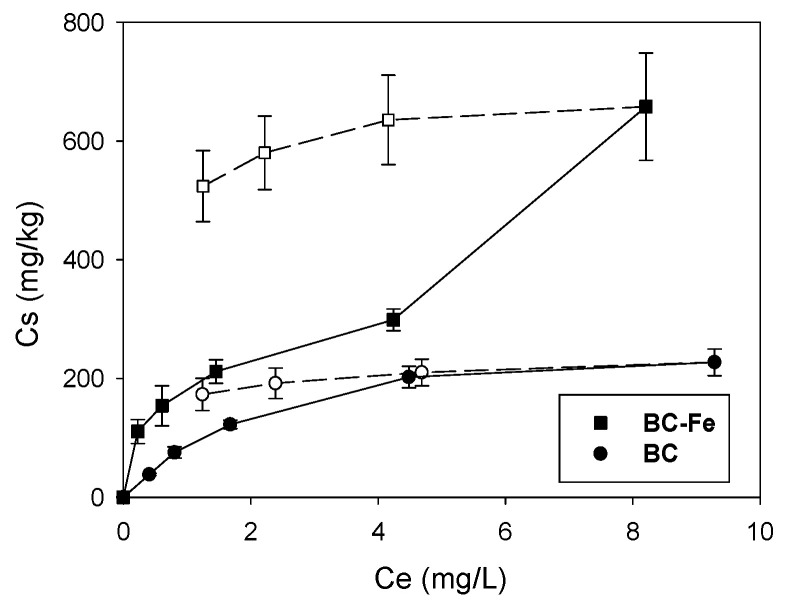
Sorption (solid lines) and desorption (dash lines) isotherms of sulfanilamide on BC and BC-Fe. Errors bars are the standard errors of triplicate samples.

The sorption isotherms of SFA on both types of biochar are shown in Figure 3 and the resulting isotherm fitting parameters are compiled in Table 2. The Freundlich model satisfactorily described the sorption isotherms with R^2^ for the isotherms greater than 0.944 (Table 2), which denotes more heterogeneous sorption surfaces where the sorption sites exhibit different energies. The isotherms indicated that, in both cases, the sorption of the antibiotic was non-linear (L-type) with calculated N_f_ values lower than 1. The activation with Fe resulted in greater non-linearity (Figure 3) (Table 2). The L-type isotherms designate that the process is initially favored when all sorption sites are available, resulting in concentration-dependent sorption [46,47]. This trend was fulfilled by both types of biochar (Figure 3 and Table 2). 

Since the isotherms were nonlinear, sorption was compared by means of the distribution coefficient K_d_ (L/kg) at an aqueous concentration (C_e_) of 0.5 mg/L (Kd_0.5_) (Table 2). Sorption coefficients Kd_0.5_ greatly increased upon activation of the biochar (from 98 to 278 L/kg). Common parameters affecting the sorption of sulfonamide antibiotics on biochar include pH, carbon content, specific surface area, porosity, and molecular size of the compound [27,48].

The pH of the solutions during sorption experiments has to be considered [49], and in this work, they ranged between 7.6 and 7.8, indicating that SFA is present mostly as a neutral molecule. Hydrophobic interactions dominated by the higher C content of BC-Fe (Table 1) would be mainly responsible for this increase in sorption rather than the pH, together with the formation of charge-assisted H-bonds [11,50,51,52]. Since an increase in sorption was observed upon activation without great variations in the specific surface area values, this increase can be attributed to the microsite modifications on BC’s surface in BC-Fe and the changes in the texture as observed from SEM micrographs (Figure 1) and data from Table 1. This was also detected for sulfamethazine on magnetite-coated biochar, promoting polar interactions through the formation of -O- moieties [51,52] or also additional cation-bridging between the iron species and biochar [36]. To discern the sorption mechanisms of SFA on BC and BC-Fe, FTIR spectra after SFA sorption were obtained (results shown in Figure S1 of the Supplementary Information). Although it is difficult to observe bands of SFA in the biochar’s FTIR spectra after sorption, likely due to the low amounts sorbed, several changes in the typical absorption bands of biochar samples were found. The main changes recorded for BC-Fe were increases in the intensity of the band registered at 872 cm^−1^ after SFA addition. This indicates the interaction with -C-H groups of the aromatic ring (π-interactions) [53]. Similarly, the initial band determined at 1430 cm^−1^, which corresponds to vibrations -C=C- of aromatic rings, was shifted towards higher wavenumbers, directly demonstrating the involvement of the aromatic moieties. The only band of SFA appearing after sorption in the FTIR spectra is that at 698 cm^−1^, which corresponded with the planar ring bending mode of the antibiotic [54] and confirms the π–π interactions between BC-Fe and SFA in agreement with previous work [52]. Finally, the 1600 cm^−1^ band of the FT-IR spectra of BC-Fe is also shifted towards lower wavenumbers. This band is attributed to aromatic and carbonyl groups of the carboxylic groups, which could explain the presence of charged-assistant H-bonds, also supported by the decrease in the intensity of the band at 1384 cm^−1^ (O-H- bending of phenols). On the contrary, the intense band at 1032 cm^−1^ (acid derivatives groups, aliphatic C-O-C, and -OH from oxygenated functional groups of cellulose and hemicellulose and methoxy groups of lignin) was not involved in the interactions between SFA and BC-Fe, for instance in the formation of this type of H-bonds, which were formed with more available functional groups. In the case of BC, lower differences were observed after SFA sorption, highlighting the increase in the vibrations -C=C- of the aromatic ring band at 1440 cm^−1^, which most likely implies interactions between the aromatic rings, since, in the BC + SFA sample, a new band appeared corresponding with -OH phenol but not hydrogen bonded [44]. Therefore, in this case, the formation of H-bonds seemed to be less important than in the activated biochar BC-Fe. 

The bioavailability and mobility of SFA in soil are controlled not only by the extent of sorption, but also by its reversibility. Desorption isotherms (Figure 3) and the calculated Thermodynamic Index of Irreversibility (TII), 0.77 and 0.79 for BC and BC-Fe, respectively (Table 2), revealed that SFA sorption is hysteretic with a more pronounced effect for BC-Fe, which is typical of organic compounds on BC [55] and of sulfonamides antibiotics on air-activated BC [27]. This is an important factor in the control of soil contamination using these compounds since the irreversible sorption would reduce the potential release later. Likewise, irreversible sorption was suggested by Luo et al. [18], who reported that the desorption of several sulfonamide antibiotics in different carbonaceous materials was plausible when sorption occurred through covalent bonds. Additionally, it is known that polar interactions promoted hysteresis to a greater extent, as compared to the partitioning mechanism [56], at the same time that the L-type isotherms imply sorption sites with different energy levels, which could have turned to incomplete desorption [57]. Furthermore, intraparticle diffusion, pore deformation during sorption, or entrapment into micropores, together with some experimental artifacts, have been described as causes of sorption irreversibility of organic compounds in BC-amended soil [58]. To confirm the results obtained, an additional experiment was performed. We directly measured the sorption of SFA on BC samples under the same conditions of sorption, that is, 20 mg of BC or BC-Fe samples (triplicate) were equilibrated separately in Pyrex^®^ glass tubes with 8 mL of an aqueous solution of SFA with an initial concentration of 1 mg L^−1^. After equilibration, 6 mL of supernatant was removed and replaced by 8 mL of a mixture of water/methanol (7/1). In this way, the amount of pesticide initially sorbed was directly determined from their desorbed amounts with the water/methanol mixture. Polar organic solvents, such as methanol, have been traditionally used to approximate the fraction of potentially bioavailable organic contaminants [59,60]. Under these conditions, only 54% of the initially added SFA was recovered from both types of biochar. This suggests that hydrophobic interactions, π–π interactions, and H-bonding observed in FTIR studies are not the only sorption mechanisms or processes occurring during the sorption of SFA. Therefore, two additional concomitant processes may also occur, such as chemical degradation after sorption on the BC surface [52] and/or irreversible sorption of SFA, which limits desorption. 

### 2.3. Dissipation Studies of SFA on Biochar

**Figure 4:**
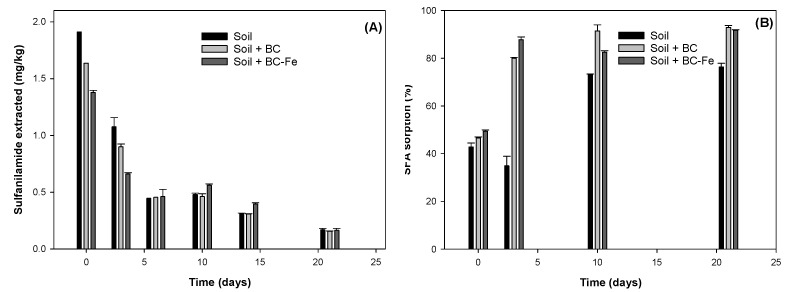
(**A**) Amount of sulfanilamide extracted in untreated soil and soil treated with BC and BC-Fe and (**B**) SFA sorption at selected times during the incubation experiment.

The dissipation kinetics of SFA in untreated soil and soil treated with BC and BC-Fe are shown in Figure 4A and the dissipation parameters are compiled in Table 3. The dissipation data satisfactorily fit first-order kinetics, with R^2^ > 0.831 (Table 3). Remarkably, similar and short half-lives were found in untreated soil and soil treated with BC, with half-lives of 4 and 4.5 days, respectively, whereas soil + BC-Fe rendered a t_1/2_ of 6.4 days (*p* < 0.05) (Table 3). It was noticeable that at t = 0, the amount of SFA extracted from untreated soil was close to the concentration initially added (96%), but in soil + BC and soil + BC-Fe, only 82% and 69% were extracted at t = 0, respectively. As aforementioned, two phenomena could immediately occur: (1) Strong sorption on BC particles, which hampered the completed extraction, or (2) abiotic degradation catalyzed by the biochar, since the extraction was conducted immediately after the SFA application to the mixtures.

The short half-lives of SFA observed in this study contrasted with the values from the literature, which reported higher persistence of sulfonamides in soils [7,61]. Several pathways have been described for the degradation of sulfonamides in soil, the most relevant being photodegradation, hydrolysis, and biodegradation [7,13,62,63]. Photocatalytic degradation of SFA in our case may have little effect since the experiments were performed in the dark. Furthermore, in general terms, it has been reported that antibiotics may enter soil pores and be fixed to soil particles receiving protection from sunlight [62]. In our study, the rapid disappearance of SFA in soil and soil treated with BC and BC-Fe could be a consequence of SFA sorption on organic or mineral particles [64] with the contribution of abiotic or biotic degradation, as suggested by the sorption experiments. For instance, Mohatt et al. [63] reported for sulfamethoxazole that its rapid transformation in soil was triggered by abiotic reactions between SFA and Fe(II) generated by the microbial reduction of Fe(III) from soil minerals, which is in agreement with our results. 

To further investigate the abiotic or biotic processes affecting the dissipation of SFA in soil and soil treated with biochar, incubation experiments using sterilized soils (Figure S2) under the same conditions were performed for 6 days. Interestingly, SFA was almost fully extracted at the beginning of the experiment (t = 0), with extraction percentages in the following order: Untreated soil (100 %) > soil + BC (93%) > soil + BC-Fe (90%) (Figure S2), in contrast to recoveries obtained under non-sterilized conditions, particularly for soil treated with BC (Figure 4). In the same line, for the rest of the sampling times, no significant differences between treatments were observed (*p* > 0.05), and the concentrations were higher in all cases than in non-sterilized conditions (*p* < 0.05). Several hypotheses can be inferred from these results. First, there is a contribution of microbial biodegradation in the dissipation of SFA in soil since higher recoveries of SFA were observed in sterilized samples in comparison to non-sterilized samples. This agrees with previous work on the biodegradation of other sulfonamides in soils [2,3,6]. On the other hand, the non-extractable fraction of SFA was assumed to be strongly bonded or abiotically degraded [2], with little contribution of BC or BC-Fe to the dissipation of SFA.

The sorption data of SFA obtained from the total amount extracted at different incubation times (Figure 4B) reveal the role of sorption during dissipation and contributes to elucidating the processes taking place in the dissipation of SFA in soil and soil treated with BC and BC-Fe. Greater SFA sorption was observed for soil treated with BC and BC-Fe when compared to untreated soil (*p* < 0.05). After 3 days of incubation, the amount of SFA sorbed reached up to 80% and 88% for soil treated with BC and BC-Fe, respectively, whereas for untreated soil, only 35% of SFA of the amount extracted was sorbed (Figure 4B). Our results show that although there are important differences in SFA sorption among treatments, these differences do not significantly affect the dissipation rate of SFA in soil. Hence, our results indicate that soil treatment with BC may not induce significant changes in the soil bioavailability of SFA. Most likely, BC and BC-Fe once in soil would interact with soil colloidal components, reducing the exposed surfaces and, hence, the increase in sorption would not increase the persistence of SFA, in contrast to previous studies with other organic compounds such as herbicides in soil amended with BC [35].

### 2.4. SFA Leaching Studies

**Figure 5:**
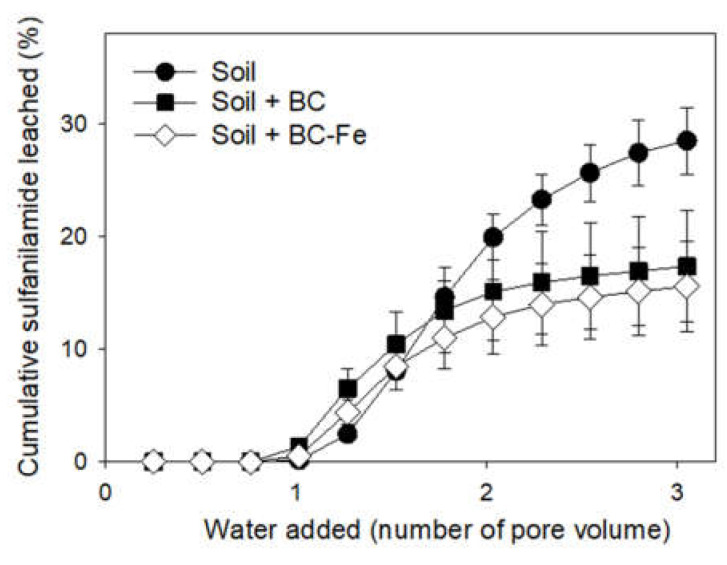
Cumulative sulfanilamide breakthrough curves in soil and soil treated with BC and BC-Fe.

The soil column leaching experiments were conducted to assess the effect of BC and BC-Fe addition on the mobility of SFA in the soil. The breakthrough curves (BTCs) of SFA in untreated soil and soil treated with BC and BC-Fe are displayed in Figure 5, and relevant data of the leaching experiment are compiled in Table 4.

Unlike the results from the dissipation experiment, the addition of BC and BC-Fe to soil affected the leaching of SFA. Accordingly, SFA leached to a greater extent in untreated soil as compared to BC-treated soils (*p* < 0.05). However, the differences between both types of biochar were not statistically significant (*p* > 0.05) (Figure 5 and Table 4). The information regarding the transport characteristics of sulfonamides in biochar-amended soil is still scarce and contradictory results have been reported, but, in general, their mobility is related to the organic matter content of the soil [5,6,65]. For instance, Vithanage et al. [65] found no temporal retardation of sulfamethazine in biochar-treated soil as compared to untreated soil, and the addition of biochar prepared at 700 °C increased the retention of this antibiotic in sandy loam and loamy sand soils, which agrees with our leaching data. On the other hand, Tang et al. [50] indicated that the transport of sulfapyridine was reduced after the addition of fresh and aged biochar to soil.

The movement of SFA throughout the soil profile was determined after the extraction of the soil columns at the end of the leaching experiment. SFA residues in untreated soil were <1% (Table 4), whereas after the addition of BC and BC-Fe to the topsoil, similar amounts of SFA were extracted with 9 and 7 % of the amount initially added, respectively (Table 4). Presumably, the greater sorption of soil treated with BC and BC-Fe observed during the incubation experiment (Figure 5) influenced the lower amount leached and extracted, although, as pointed out above, differences were not great enough to detect significant differences between the biochar, suggesting again that the modifications in the surface of BC are blinded in the bulk soil. This could be a result of the surface plaque or coatings that are observed from soil-recovered weathered biochar [41,42]. However, these results demonstrated that biochar, and particularly its modification with dissolved Fe, could reduce the transport of SFA under specific conditions. Lower availability for leaching of the antibiotic in soil could be attributed to enhanced and irreversible sorption and/or degradation as compared to untreated soil [66]. As pointed out above, there were no correlations between sorption, dissipation and leaching. Incongruities between these factors have been traditionally endorsed to the dynamic and saturated conditions in the leaching experiment compared with the static conditions in dissipation and sorption experiments [35,65,67,68], together with different anaerobic/aerobic conditions which can also alter the ability of the specific microorganisms as well as abiotic reactions to degrade compounds [69].

## 3. Materials and Methods

### 3.1. Antibiotic, Soil, and Biochar

Sulfanilamide (SFA), selected as an example of sulfonamide-type antibiotics (Figure S3), was purchased from Sigma-Aldrich (Madrid, Spain) (analytical purity > 99%). SFA has a water solubility of 7500 mg/L 25 °C and has two pK_a_ at 3.22 and at 10.6 [48]. The solutions used in laboratory experiments were prepared from a methanolic stock solution of 200 mg/L of SFA.

The soil used in this study was a sandy clay loam soil located at the IRNAS-CSIC experimental field in Coria del Río, Southern Spain (37°16′54″ N, 6°3′54″ W). Soil analysis was performed in the Soil Analysis Service from IRNAS-CSIC and revealed 59% sand, 18% silt, and 23% clay; organic carbon of 0.65%; CaCO_3_ of 3.7%; <2% in amorphous Al and Fe oxides with a measured pH of 7.7 determined in a distilled water slurry (1:2.5; *w*/*v*).

Commercial biochar (BC) produced at 500 °C from oak hardwood (*Quercus ilex*) was provided by Vermichar, Lombricompost S.L. (Spain). The activation consisted of a suspension of BC (10 g) in contact for 24 h (at 25 °C) with a solution of 5 g of FeNO_3_ dissolved in 40 mL of distilled water (0.52 M). Then, the biochar (BC-Fe) was washed three times with 100 mL of DI water and dried at 60 °C overnight. 

The characterization of BC samples was performed by different techniques. A combustion elemental analyzer (LECO, Model CHN 932, Okehampton, Devon, UK) was used to determine the elemental analysis of the samples, and the BET surface area of samples was obtained via physisorption of N_2_ at 77 K (ASAP Micromeritics Model 2010, Norcross, GA, USA). The t-plot and BJH (Barret–Joyner–Halenda) methods were used to determine the micro-and mesopore volumes of the samples, respectively. Water slurries of the biochar in mixtures of 1:2.5 (*w*:*v*) served to establish the pH. The Fourier-transform infrared (FTIR; Bruker Invenio-X, Akbou, Algeria) spectra for the region between 4000 and 400 cm^−1^ were obtained for the analysis with samples prepared at 1% (*w*/*w*) in KBr. The Fe content in the biochar samples was determined by inductively coupled plasma source mass spectrometry, ICP-MS (Agilent 7800 model, Waltham, MA, USA), after the acid digestion of the samples (100 mg) with 1:3 mixture of concentrated nitric and hydrochloric acids (aqua regia). Biochar was imaged using and FEI-TENEO (Waltham, MA, USA) scanning electron microscope (SEM) to visually examine their physical structure. The samples were coated with a 10 nm layer of P to reduce sample charging and improve image resolution.

### 3.2. Sorption Experiments of SFA on Biochar

SFA sorption isotherms on both types of biochar were performed using the batch equilibration method. In triplicate, 20 mg of BC or BC-Fe were weighed in Pyrex^®^ glass tubes and equilibrated for 24 h with 8 mL of different aqueous solutions of SFA with initial concentrations ranging from 0.5 to 20 mg/L at 25 ± 2 °C. Then, the tubes were centrifuged, and 4 mL of supernatant was removed, filtered (0.45 μm pore size), and analyzed by HPLC according to Section 3.5. A corresponding set of controls without BC was set up to document possible losses due to degradation or sorption on tube walls. Immediately after the sorption from the point of highest concentration (20 mg/L), 4 mL of water was added to the tubes and the mixtures were resuspended and shaken overnight. The samples were centrifuged, and 4 mL of the supernatant solutions were removed, filtered, and analyzed by HPLC. This process was repeated three times to achieve the desorption isotherm.

### 3.3. Dissipation Kinetic of SFA in Soil and Treated Soil

An incubation experiment was designed to assess the dissipation kinetics of SFA in untreated soil and soil treated with BC and BC-Fe at 2% (*w*/*w*). In duplicate, glass jars with screw caps were filled with untreated soil samples or treated soil (100 g). The water content was adjusted at ~30 % and SFA was applied in a dose of 2 mg/kg by adding 4 mL of an aqueous solution of 50 mg/L. The soil samples were placed in an incubator at 25 ± 2 °C in darkness and aerobic conditions for 21 days. Subsamples of 3 g of soil were periodically sampled 0, 3, 6, 10, 14, and 21 days after treatment (DAT) in triplicate with a sterilized spatula and frozen until analysis by HPLC. The extraction to determine potential bioavailable SFA residues consisted of adding 8 mL of a mixture of water/methanol (7/1) and shaking for 24 h. Then, the samples were centrifuged (8000 rpm) and supernatants were filtered (syringe filter; 0.45 µm) and analyzed by HPLC. 

To ascertain whether the soil addition of BC and BC-Fe induces modifications in the easily available fraction of SFA during its dissipation in the soil, we simultaneously determined SFA sorption during the incubation experiment at selected times coinciding with sampling times established (t = 0, 3, 10 m and 21 days) following a methodology previously described by our group with other compounds [35,55,70]. In this case, 5 g of both untreated and treated soil were taken from the incubation jars and weighed in specific centrifugal tubes (Macrosep^®^ Advance from Pall Corporation, Hampshire, UK) with a membrane of polyethersulfone (0.45 μm). Then, the tubes were centrifuged at 10,000 rpm for 10 min to obtain the soil solutions and quantify the aqueous concentration of SFA by HPLC. The difference between the total amount of SFA extracted in the previous step and the amount of SFA in the soil solution was assumed to be adsorbed, and the percentage of adsorption was calculated. 

### 3.4. Leaching of SFA in Soil and Treated Soil

The downward mobility of SFA through the soil profile after the simulation of rainfall episodes was established in a leaching experiment using soil glass columns 30 cm long and an internal diameter of 3.1 cm. The column bottoms were filled with glass wool plus 10 g of sea sand. Then, 160 g of dried soil was packed within the 0–20 cm of the soil columns. The top first 5 cm of soil (~40 g of soil) was treated at 2 % *w*/*w* with BC and BC-Fe. The design was completed by placing 10 g of sea sand on the top of the columns. The experiment was performed in triplicate. The columns were saturated after the application of 100 mL of DI water and drained for 24 h, resulting in soil column pore volumes of 61 ± 1 mL. Then, 3 mL of an aqueous solution of SFA at 50 mg/L (0.15 mg) was added to the top of the column (2 mg/kg for the first 10 cm of soil), similar to the concentration used in the incubation experiment. Twice a day, distilled water was added simulating rainfall events of 20 mm (20 L/m^2^) to the top of columns in an amount equivalent to 3 pore volumes. Soil leachates were collected in 25 mL-vials, stored, and kept at 4 °C until HPLC analysis. At the end of the experiment, soil columns were extracted with water/methanol (7/1) to quantify the remained residues of SFA in the soil.

### 3.5. Sulfanilamide Analysis

A Waters 600E chromatograph (Santa Clara, CA, USA) coupled to a Waters 996 detector and Kinetex C18 column (Phenomenex) was used to quantify the aqueous concentration of SFA in the solution. The analytical conditions were a flow rate of 1 mL/min, 25 µL injection volume, a mobile phase of 95:5 (*v*/*v*) water:methanol, and UV detection at 257 nm, reaching a retention time of 3.4 min. An example chromatogram of SFA at 2 mg/L in an aqueous solution is shown in the SI (Figure S4).

### 3.6. Data Treatment

Sorption isotherms were fitted to the linearized form of the Freundlich equation.

log C_s_ = log K_f_ + N_f_ logC_e_ (1)

where C_e_ is the aqueous concentration of SFA (mg/L) in solution, C_s_ is the amount of antibiotic sorbed (mg/kg), K_f_ is the sorption coefficient, and N_f_ (1/n) is the nonlinearity factor. Distribution coefficients (K_d_) were also calculated to discern the extent of the sorption process in an aqueous solution.

The extent of the desorption process was assessed by the calculation of the Thermodynamic Index of Irreversibility (TII) following the equation: TII = 1 − N_fd_/N_f_, where N_f_ and N_fd_ are the Freundlich constants obtained from the sorption and desorption isotherms, respectively [71]. Distribution coefficients, K_d_ (L/kg), were also calculated to discern the extent of the sorption process in an aqueous solution, calculated as C_s_/C_e_.

Dissipation curves were fitted to the first-order kinetics:

C = C_0_ e^−kt^ (2)

where C (mg/kg) and C_0_ (mg/kg) are the concentrations of sulfanilamide extracted in the soil at time t (days) and t = 0, respectively, and k (day^−1^) is the first-order dissipation constant. Half-life (t_1/2_) was computed from the dissipation constant using the equation:

t_1/2_ = (ln 2)/k (3)

Statistical analyses were performed with Sigmaplot version 14.5 (Berlin, Germany) for Windows. The data obtained were compared using one-way ANOVA and pairwise comparisons by the application of Tukey’s test. Significant differences were established at a level of probability *p* < 0.05.

## 4. Conclusions

Our results indicate that the modification at the microsite scale of a BC with Fe is responsible for the enhanced sorption observed for the antibiotic sulfanilamide as compared to the pristine BC material. Since greater changes in SSA were not observed, the main sorption mechanisms confirmed by FTIR studies were π–π interactions and H-bonds, with the latter being more important in BC-Fe. Nevertheless, in both cases, a significant fraction of SFA was irreversibly bonded and/or chemically degraded. The activation of BC with Fe did not give rise to a longer residence time of the antibiotic in soil. Both BC and BC-Fe reduced the movement of sulfanilamide in soil to a comparable extent. Accordingly, the activated biochar, when added to soil, could interact with soil colloidal components, reducing the exposed modified surfaces and resulting in similar behavior to that of the inactivated biochar. Our results demonstrated that the Fe-activation of biochar could be a useful strategy to increase the sorption of SFA for water removal, but not for soil. Further investigations are needed to deepen insights into the interactions of activated biochar with soil components with the aim to control contamination derived by the presence of veterinary drugs in soils. Processes and alterations of the biochar surfaces once in soil at different scales remain unknown and are even more convoluting when interactions with dissolved metal cations are considered.

## Figures and Tables

**Table 1:** Selected properties of BC and BC-Fe.

Biochar	C (%)	H (%)	O (%) ^1^	N (%)	Ash (%) ^2^	Fe (g/kg)	SSA (m^2^/g)	V_tot_ (cm^3^/g)	V_mic_ (cm^3^/g)	V_meso_ (cm^3^/g)	pH ^3^
BC	39.1	1.2	17.5	0.6	12	2.7	2.20	0.01300	0.00056	0.00727	7.9
BC-Fe	46.6	1.4	31.8	0.9	34	43.9	4.13	0.01051	0.00452	0.00320	5.6

^1^ Determined at 540 °C. ^2^ Calculated assuming less than 1% of S without ash content. ^3^ Measured in (1/5) water slurries.

**Table 2:** Freundlich coefficients for sulfanilamide isotherms on BC and BC-Fe.

Treatment	K_f_	N_f_	R^2^	K_fd_	N_fd_	R^2^	TII ^1^	Kd_0.5_ (L/kg) ^2^
BC	77 (68–87) ^3^	0.64 ± 0.07 ^4^	0.953	169 (167–171)	0.135 ± 0.007	0.996	0.79	98
BC-Fe	201 (178–228)	0.57 ± 0.02	0.997	76.9 (68.2–86.7)	0.122 ± 0.021	0.944	0.77	278

^1^ TII: Thermodynamic index of irreversibility; TII = 1 − N_fd_/N_f_. ^2^ K_d_ = C_s_/C_e_ at C_e_ = 0.5 mg/L. ^3^ Values in parentheses correspond to the standard error about the Freundlich coefficients. ^4^ value ± standard error.

**Table 3:** Single first-order dissipation constants and half-lives for SFA in untreated and soil treated with BC and BC-Fe.

	k (d^−1^)	t_1/2_ (d)	R^2^
Soil	0.172 ± 0.029 ^1^	4.0	0.950
Soil + BC	0.153 ± 0.026	4.5	0.945
Soil + BC-Fe	0.109 ± 0.031	6.4	0.831

^1^ value ± standard error.

**Table 4:** Summary of leaching data extracted from the relative and cumulative breakthrough curves (BTCs) of SFA in untreated soil and soil treated with BC and BC-Fe. Different letters in each column indicate significant differences in values (*p* < 0.05).

Treatment	C_max_ (mg/L) ^1^	Total Leached (%)	Total Extracted (%)	Total Not Recovered (%)
Soil	0.78 ± 0.03 ba	31 ± 1 a	<1	69
Soil + BC	0.61 ± 0.05 a	19 ± 1 b	9 ± 3	72
Soil + BC-Fe	0.45 ± 0.11 b	16 ± 4 b	7 ± 2	77

^1^ Maximum concentration of SFA in leachates.

## Data Availability

The data presented in this study are available on request from the corresponding author.

## Supplementary Materials

The following supporting information can be downloaded at: https://www.mdpi.com/article/10.3390/molecules27217418/s1, Figure S1. FT-IR spectra of sulfanilamide, BC, and BC after sorption of SFA (A) and spectra of sulfanilamide, BC-Fe, and BC-Fe after sorption of SFA. Figure S2. Amount of sulfanilamide extracted in untreated soil and soil treated with BC and BC-Fe under sterilized conditions. Figure S3. Chemical structure of the antibiotic sulfanilamide. Figure S4. Chromatogram of SFA in aqueous solution (C = 2 mg/L).

## Sample Availability

Samples of the compounds are not available from the authors.
