# Supplementary material for: The Role of Nanoengineered Biochar Activated with Fe for Sulfanilamide Removal from Soils and Water
Source: Molecules. 2022 Nov 1;27(21):7418. doi: 10.3390/molecules27217418 (PMC9657491; doi:10.3390/molecules27217418)
Supplement: Supplementary file 1 [file molecules-27-07418-s001.zip › molecules-1968597-supplementary.pdf]

Supplementary Material

The Role of Nanoengineered Biochar Activated with Fe for Sulfanilamide Removal from Soils and Water

Beatriz Gámiz,*[1] Pilar Velarde[1], Kurt A. Spokas[2], Lucía Cox[1]

[1] *Instituto de Recursos Naturales y Agrobiología de Sevilla (IRNAS), CSIC, Avenida Reina Mercedes 10, 41012 Sevilla, Spain*

[2] *U.S. Department of Agriculture, Agricultural Research Service, 439 Borlaug Hall, 1991 Upper Buford Circle, St. Paul, Minnesota 55108, United States*

***Corresponding Author:** Dr. Beatriz Gámiz

ORCID: 0000-0002-7244-778X

Address: Instituto de Recursos Naturales y Agrobiología de Sevilla (IRNAS), CSIC

Avenida Reina Mercedes 10

41012 Sevilla, Spain

Phone: +34 954624711

E-mail: bgamiz@irnase.csic.es

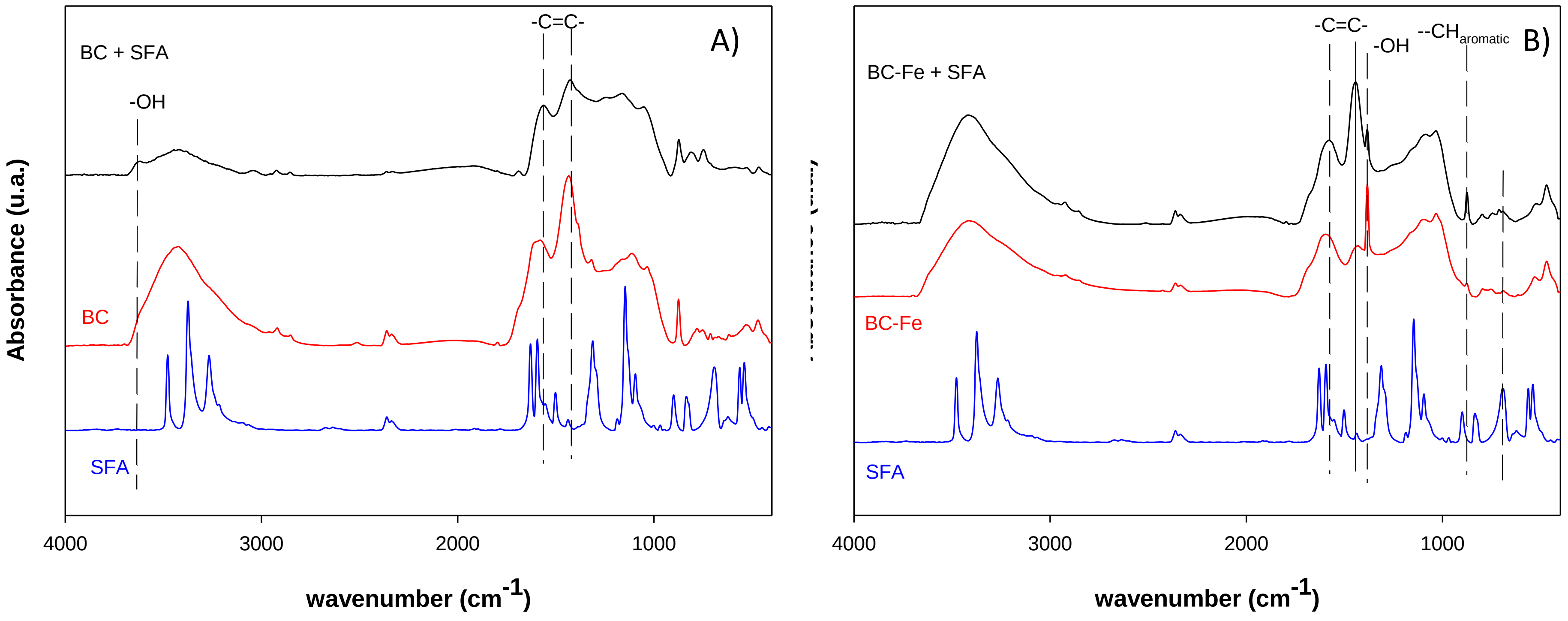

Figure S1. FT-IR spectra of sulfanilamide, BC and BC after sorption of SFA (A) and spectra of sulfanilamide, BC-Fe and BC-Fe after sorption of SFA.

.

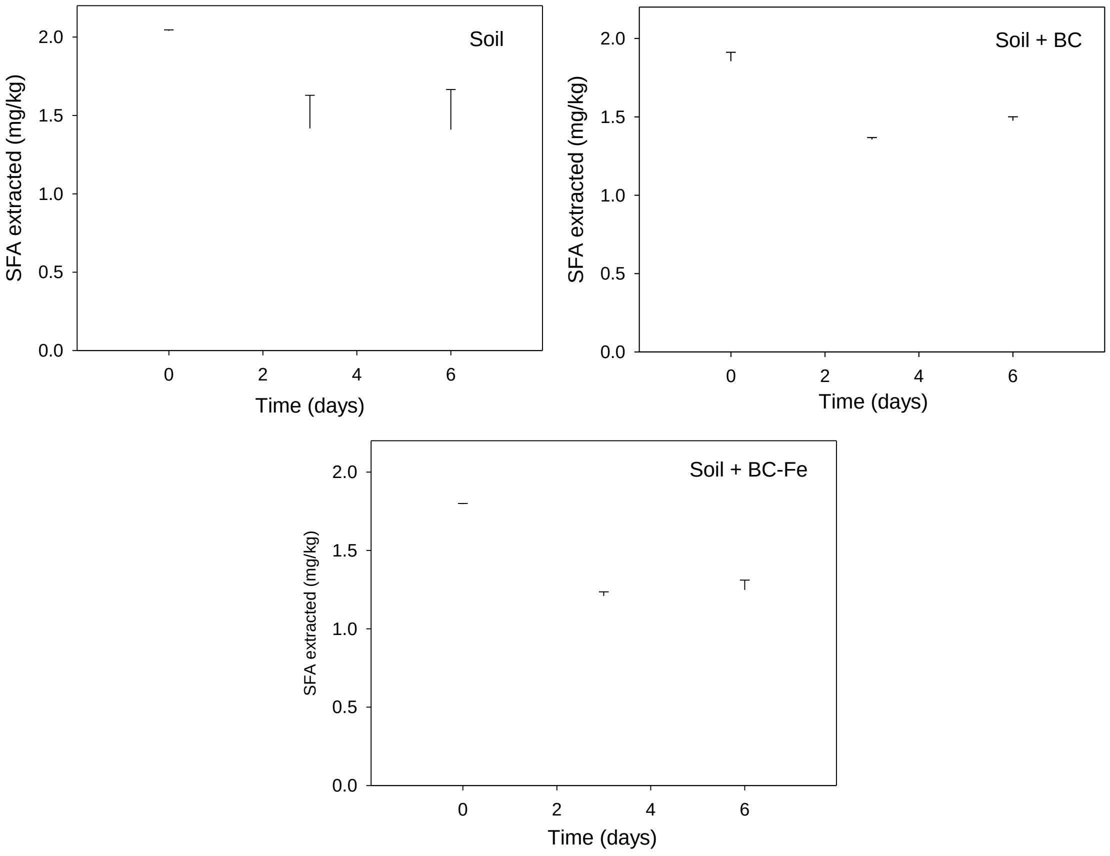

Figure S2. Amount of sulfanilamide extracted in untreated soil and soil treated with BC and BC-Fe under sterilized conditions.

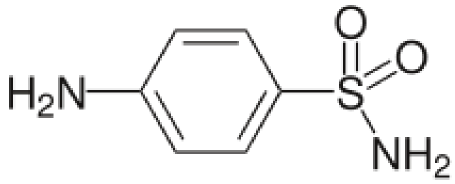

Figure S3. Chemical structure of the antibiotic sulfanilamide.

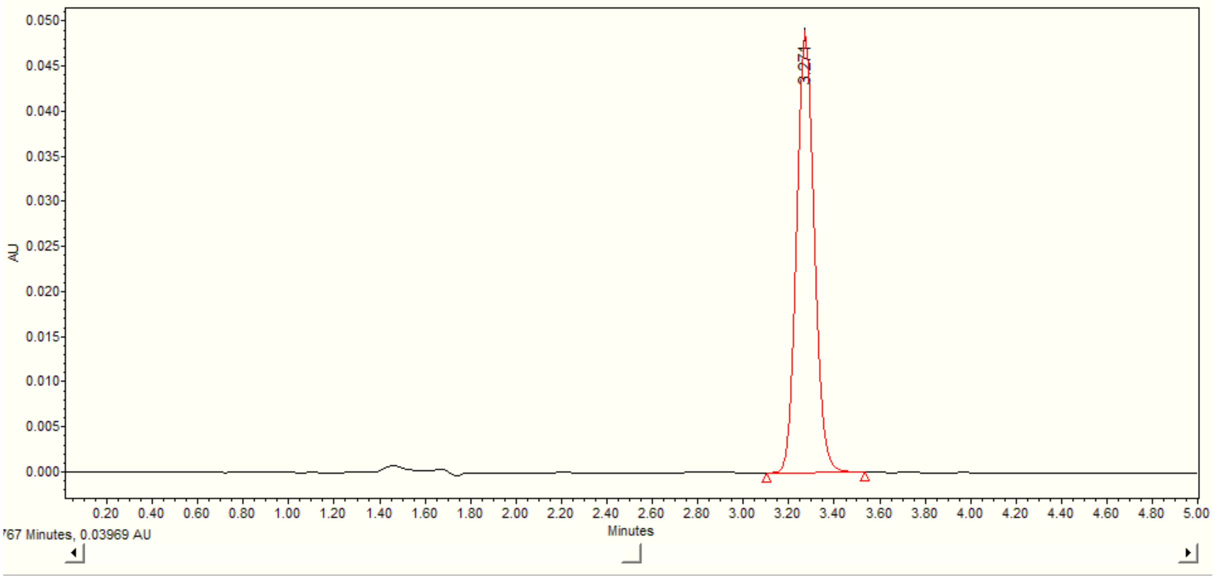

Figure S4. Chromatogram of SFA in aqueous solution (C= 2 mg/L).
